# Stem cell mTOR signaling directs region-specific cell fate decisions during intestinal nutrient adaptation

**DOI:** 10.1126/sciadv.adi2671

**Published:** 2024-02-09

**Authors:** Jaakko Mattila, Arto Viitanen, Gaia Fabris, Tetiana Strutynska, Jerome Korzelius, Ville Hietakangas

**Affiliations:** ^1^ Faculty of Biological and Environmental Sciences, University of Helsinki, Helsinki 00790, Finland.; ^2^ Institute of Biotechnology, University of Helsinki, Helsinki 00790, Finland.; ^3^ School of Biosciences, University of Kent, Canterbury CT2 7NJ, UK.

## Abstract

The adult intestine is a regionalized organ, whose size and cellular composition are adjusted in response to nutrient status. This involves dynamic regulation of intestinal stem cell (ISC) proliferation and differentiation. How nutrient signaling controls cell fate decisions to drive regional changes in cell-type composition remains unclear. Here, we show that intestinal nutrient adaptation involves region-specific control of cell size, cell number, and differentiation. We uncovered that activation of mTOR complex 1 (mTORC1) increases ISC size in a region-specific manner. mTORC1 activity promotes Delta expression to direct cell fate toward the absorptive enteroblast lineage while inhibiting secretory enteroendocrine cell differentiation. In aged flies, the ISC mTORC1 signaling is deregulated, being constitutively high and unresponsive to diet, which can be mitigated through lifelong intermittent fasting. In conclusion, mTORC1 signaling contributes to the ISC fate decision, enabling regional control of intestinal cell differentiation in response to nutrition.

## Teaser

mTORC1 signaling regulates stem cell size and directs absorptive versus secretory cell fate upon intestinal nutrient adaptation.

## INTRODUCTION

Nutrient intake modulates the physiology of adult animals not only through alteration of metabolism of tissues but also through changes in cellular composition of organs through the activity of somatic stem cells. For example, the net volume and morphology of the small intestine are strongly regulated by nutrition to match the organ’s absorptive, metabolic, and signaling functions with the physiological needs of the animal (*1*–*3*). This includes coordinated control of proliferation and differentiation of stem cells and size of differentiated cells (*2*–*4*). How the balance between these parameters is dynamically controlled by nutrient signaling in the spatial context of organs, for example, in different intestinal regions, remains poorly understood.

The *Drosophila* midgut, the counterpart of mammalian small intestine, contains four distinct cell types: large absorptive enterocytes (ECs) and their precursors enteroblasts (EBs), as well as the small secretory enteroendocrine (EE) cells, all differentiating from the mitotic intestinal stem cells (ISCs) (*5*). The EB/EC and EE differentiation is determined by the strength of Notch signaling. ISCs with high Delta expression direct their daughter cells toward EB/EC fate, while ISCs with low Delta promote EE differentiation (*6*, *7*). Feeding of experimental diet with high cholesterol reduces Delta-Notch signaling, promoting ISC differentiation toward the EE cell fate (*8*). However, whether the adaptive growth of the intestine upon transition from fasted to fed state (*2*) involves differential control of EB/EC and EE fates remains to be addressed. The ISC differentiation toward the EB/EC lineage involves a prominent increase in cell size, which is mediated by Notch-dependent activation of mTORC1 in EBs, driving EB differentiation toward the EC fate (*9*).

Midgut size is highly adaptive to nutrition: When calorie-restricted, ISC proliferation is low and the midgut volume decreases through EC loss and size reduction (*2*, *4*). Upon feeding, the proliferation and differentiation of ISCs is increased, and the ECs gain in size, leading to increase in midgut volume (*2*, *4*, *10*, *11*). The nutrient-induced ISC proliferation depends on insulin-induced phosphatidylinositol 3-kinase (PI3K)/Akt signaling (*2*, *11*), while the EC size increase upon feeding requires mammalian Target of Rapamycin complex 1 (mTORC1) activity (*4*). Both mammalian and *Drosophila* intestines are highly regionalized organs (*12*–*14*). The *Drosophila* midgut is divided into six (R0 to R5) anatomically recognizable regions with characteristic gene expression patterns and cellular content (*12*, *14*–*16*). The regulation of ISCs is spatially defined, as evidenced by region-specific enrichment of ISC daughter cells upon DSS-induced tissue damage response (*16*). However, our current understanding on the regional specificities of nutrient regulation of intestinal cells remains very limited.

The mTORC1 is a key regulator of nutrient-induced growth in differentiated cells and tissues (*17*). However, the role of mTORC1 signaling in somatic stem cells is complex and context-dependent. Several lines of evidence imply that sustained mTORC1 signaling impairs stem cell function. In epithelial stem cells, constitutive mTORC1 activation by Wnt signaling leads to increased growth of hair follicles and disappearance of the epidermal stem cell compartment (*18*). In *Drosophila* ISCs, high mTORC1 activity upon Tuberous sclerosis complex 1/2 (TSC1 and TSC2) loss of function (LOF) leads to massively increased cell size but inhibits proliferation and differentiation (*19*). Moreover, aging increases the size of hematopoietic stem cells, impairing their function, which can be rescued by inhibition of mTORC1 signaling (*20*). In contrast to sustained mTORC1 activity, short-term stimulation of mTORC1 signaling does not compromise stem cell function and is involved in their physiological regulation. In quiescent *Drosophila* ISCs, mTORC1 activity is inhibited by high expression of TSC2 (*9*). Upon tissue damage–induced regeneration, mTORC1 signaling is transiently activated in the ISCs, which is necessary for ISC proliferation and consequent intestinal regeneration (*21*). Repeated cycles of ISC mTORC1 activation, however, lead to stem cell loss (*21*). In addition, mTORC1 is necessary to reactivate mouse muscle stem cells from quiescence after injury (*22*). In the intestine of nutrient-restricted mice, mTORC1 signaling in neighboring Paneth cells is inhibited, enhancing ISC function (*23*). How the nutrient-dependent mTORC1 signaling influences the proliferation and lineage differentiation decisions in adult stem cells remains poorly understood.

Here, we use an organ-wide approach to quantitatively analyze the regulation of cell size, number, and identity upon nutrient-induced tissue adaptation of the *Drosophila* midgut. Our data reveal a notable regional heterogeneity in the regulation of EC growth, as well as ISC differentiation by nutrient-induced cues. The number of EBs and EE cells increases upon acute transition from fasting to feeding but with contrasting regional distributions. We also observed an mTORC1-mediated increase in ISC size, which is particularly strong in the regions with nutrient-induced EB accumulation. Consistent with the regional distributions of EB and EE cells, ISC mTORC1 activity promotes the formation of asymmetric ISC-EB cell pairs while inhibiting the differentiation toward EE cell lineage. ISC mTORC1 activity promoted high expression of Delta, which is known to facilitate EB differentiation. In aged animals, the intestine responds poorly to feeding and displays abnormally elevated ISC mTORC1 activity even upon fasting. This aging-induced deregulation of intestinal nutrient adaptation and ISC nutrient sensing can, however, be mitigated by lifelong intermittent fasting. Collectively, our data demonstrate that stem cell mTORC1 activity can act as a fate determinant to mediate region-specific differentiation patterns upon intestinal nutrient adaptation with relevance to aging-associated tissue decline.

## RESULTS

### Organ-wide analysis reveals regional heterogeneity of midgut adaptive growth

**Fig. 1.**
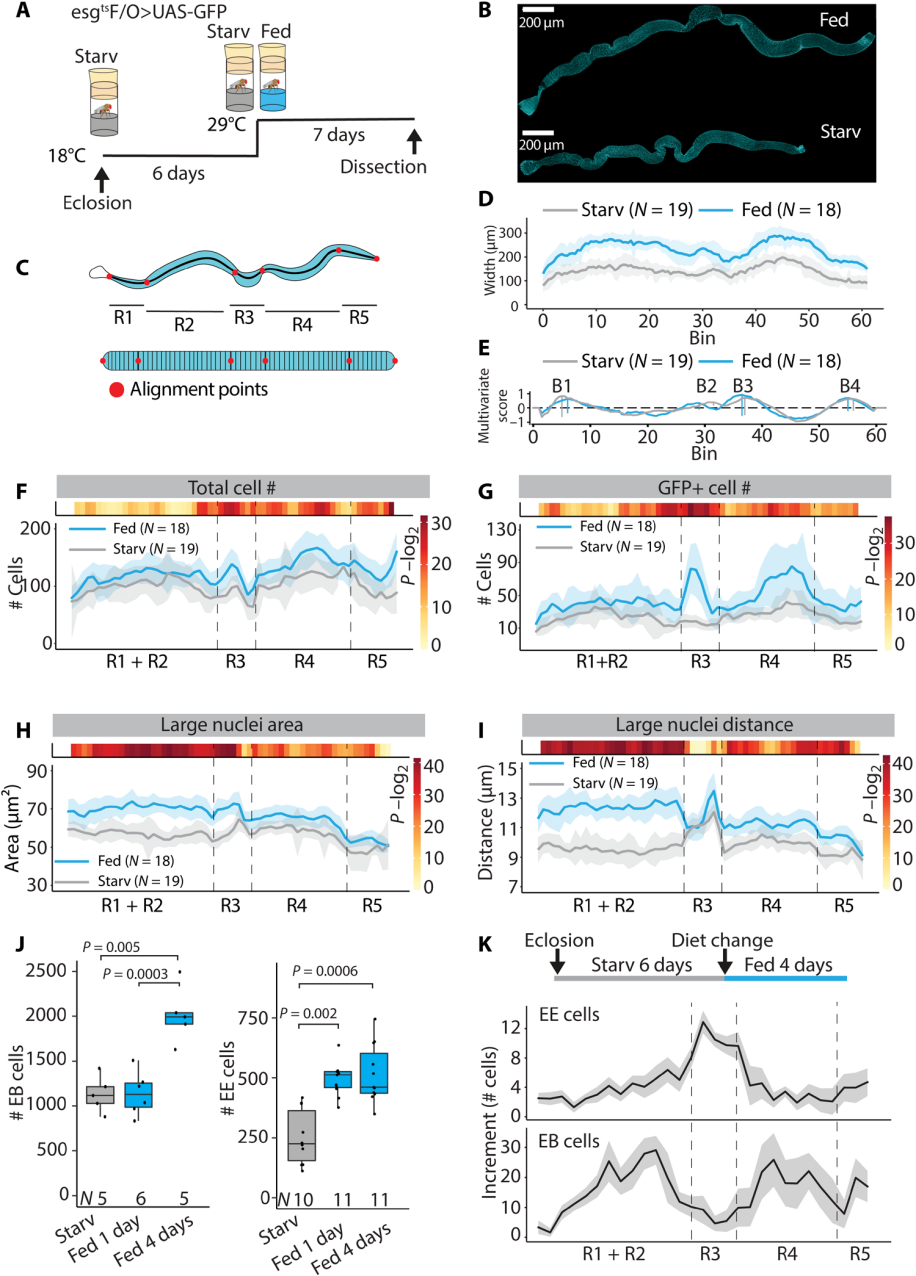
Midgut nutrient adaptation is regionally defined. (**A**) Experimental design used to obtain data for (**B** to **I**). Age-matched, mated esg^ts^F/O>UAS-GFP females were kept at +18°C for 6 days and then shifted to the permissive temperature (+29°C) for additional 7 days on either starvation (Starv) or holidic diet. (B) Representative images of DAPI (cyan)–stained midguts from experiment depicted in (A). (C) Principle of LAM. LAM transforms image-derived cellular data from three-dimensional midguts into a linearized representation, binning it into segments along the Anterior/Posterior (A/P) axis. As a result, LAM allows region to region midgut comparison. (D) Feeding results in regionally uniform midgut growth. Width profile of starved and fed midguts along the A/P axis. (E) Region border analysis shows little variation between starved and fed female midguts. The marked borders from left to right are B1, B2, B3, and B4. The multivariate border score is summed from weighted deviations of multiple variables, such as midgut width and nuclear distances. [(F) to (I)] Feeding induces regionally distinct cellular patterns. Total cell counts (F), GFP^+^ cell counts (G), large nucleus area (H) and nearest distance between large nuclei (I) along the A/P axis of starved and fed female midguts. Dashed lines indicate the main region borders. Light blue/gray shading is the standard deviation. (**J** and **K**) Feeding induces temporally and spatially defined increase in EE and EB cells. (J) Cell counts of EB and EE cells in starved, 1-day–fed, and 4-day–fed midguts. (K) Increment of EE and EB cell numbers along the midgut A/P axis 4 days after commencement of feeding. *P* values in (F) to (I) were obtained by Wilcoxon rank sum test using continuity and false discovery rate (FDR) correction (FDR < 0.05). *P* values in (J) were obtained by Wilcoxon rank sum test with multiple testing correction (FDR < 0.05). See also fig. S1.

Transition from fasted to fed state promotes ISC proliferation and EC growth, enabling dynamic adjustment of adult midgut size (*2*, *4*, *24*). Current knowledge on midgut growth regulation relies on local quantifications of distinct midgut regions, lacking the global organ-wide insight to address a possible role for region-specific regulation. Consistent with previous findings, feeding of flies on complete holidic diet after fasting on a highly restricted diet (2% sucrose) stimulated midgut growth and increased total cell numbers on both sexes (Fig. 1, A and B, and fig. S1, A to F). To achieve organ-wide insight into this adaptive growth regulation, we used the recently developed image analysis method, Linear Analysis of Midgut (LAM), that allows region-specific analysis of cellular parameters of the intestine (Fig. 1C) (*16*). Refeeding led to >1.5-fold increase in midgut width and significantly increased lengths of R2 and R4 regions (Fig. 1D and fig. S1G) while largely retaining the morphology of the regional boundaries (Fig. 1E), allowing us to align the intestinal regions of midguts with distinct size.

Despite the relatively uniform increase in midgut width, quantitative analysis of specific cellular parameters uncovered notable regional variation in the mode of nutrient regulation. An increase in total cell numbers was observed in specific areas of the central and posterior regions, while R1 and R2ab regions displayed limited increase in cell numbers (Fig. 1F). Consistently, an increase in stem cell activity, as measured by the green fluorescent protein (GFP)–marked cells in esg^ts^ Flp-Out (esg^ts^F/O) clones (*25*), was highest in the regions with increased cell numbers, including anterior R3 (copper cell region) and in R4bc, whereas the anterior regions were less affected (Fig. 1G). In contrast, EC size was most increased in the R1 and R2 regions (Fig. 1, H and I). As a surrogate to cell size, we measured the maximum nucleus cross-sectional area (hereafter referred as nuclear area) from midguts of starved and fed flies. The nuclear area correlates well with cell size and is amenable to automated quantification from three-dimensionally segmented images of 4′,6-diamidino-2-phenylindole (DAPI)–stained nuclei (fig. S1, G and H). Elimination of stem cells by Reaper overexpression revealed that the adaptive intestinal growth can occur near normally, consistent with earlier findings (fig. S1, J to O) (*4*).

Next, we wanted to address in detail the regional increase in cell numbers. Feeding induced specific changes in the numbers of ISC daughter cells, i.e., EBs and EE cells. While the number of both cell types was increased upon feeding, the response occurred through distinct kinetics. EE cell numbers increased after 1 day of feeding, while EBs increase 4 days after feeding (Fig. 1J). Notably, we observed opposite regional pattern in the distribution of EB and EE number increase. While EE cell numbers increased mainly in R3 and the region borders flanking R3, EB numbers were mostly elevated in R2 and R4 regions (Fig. 1K). Collectively, our organ-wide analysis uncovered regional differences in cell-type composition and ISC proliferation and/or cellular turnover as response to nutrient availability in the midgut. These changes are summarized in table S1.

### Region-specific mTORC1 activation controls ISC size upon feeding

**Fig. 2.**
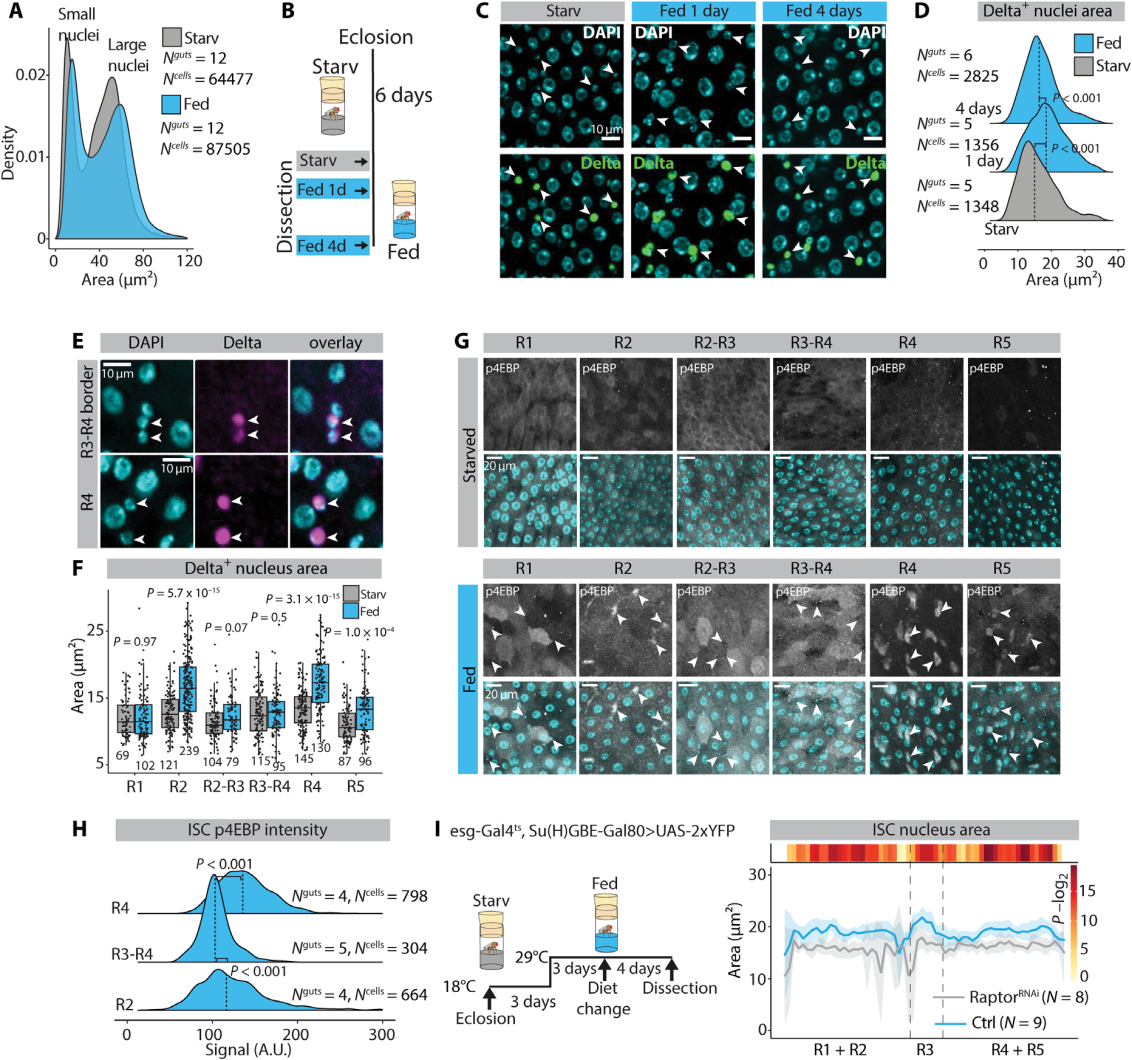
ISC size is regulated regionally by feeding-induced mTORC1 signaling. (**A**) Nucleus area distribution in starved versus fed female midguts. (**B**) Experimental design used to obtain data for (**C** and **D**). Age-matched, mated females harboring the Delta-LacZ marker were aged for 6 days at +25°C in starvation and then shifted to the holidic diet for an additional 1 or 4 days. [(C) and (D)] The size of Delta^+^ nuclei are temporally regulated in midguts of fed flies. (C) Representative images from midgut R4b region stained with α-β-galactosidase (green) and DAPI (cyan). Arrowheads point to Delta^+^ nuclei. (D) Quantification of Delta^+^ nucleus area from the experiment depicted in (B) and (C). Dashed lines indicate medians. (**E** and **F**) ISC size is regulated regionally by feeding. (E) Representative images from midguts stained with α-β-galactosidase (magenta) and DAPI (cyan). Arrowheads point to Delta^+^ nuclei. (F) Quantification of Delta^+^ nucleus area from starved and fed midgut regions. Pooled data from *N*^starved^ = 3 and *N*^fed^ = 3 midguts. *N*^cells^ are indicated in the figure panel. (**G** and **H**) mTORC1 activation by feeding is region- and cell-type–specific. (G) Representative images of midgut regions from flies kept in either starvation or holidic diet and stained with α-p4EBP (gray) and DAPI (cyan). The arrowheads point to small nuclei cells. Experimental design as in Fig. 1A. (H) Quantification of α-p4EBP intensity from Delta^+^ esg^+^ ISCs. Dashed lines indicate medians. A.U., arbitrary units. (**I**) Delta^+^ esg^+^ ISC nucleus area along the midgut A/P axis of control and Raptor-RNAi driven by esg-Gal4^ts^, Su(H)GBE-Gal80. *P* values in (D) and (H) were obtained by Kruskal-Wallis test followed by Wilcoxon test. *P* values in (F) were obtained by Wilcoxon rank sum test with multiple testing correction (FDR < 0.05). *P* values in (I) were obtained by Wilcoxon rank sum test using continuity and FDR correction (FDR < 0.05). See also fig. S2.

Organ-wide analysis of the size profiles of all intestinal cells showed that feeding-induced growth is pervasive, as it can be observed at the level of the whole population of polyploid ECs (Fig. 2A). Unexpectedly, the global analysis of cellular size distributions also revealed a size increase of the small diploid cells, which include the ISCs (Fig. 2A). Therefore, we specifically analyzed the feeding-induced size regulation of ISCs by using the ISC marker Delta-LacZ (Fig. 2, B and C) (*26*). Notably, ISC size increased significantly upon feeding, being most prominent already at the 1-day time point (Fig. 2, C and D). Thus, ISC enlargement preceded the increase in EB cell numbers (Fig. 1J). Next, we analyzed whether the ISC size is regionally regulated upon changing nutrient intake. Nutrient-induced ISC growth was highly regionalized: ISCs at R2, R4, and R5 displayed feeding-induced size increase, while ISC size in R1 as well as in the R2-R3 and R3-R4 border regions was unresponsive to nutrient availability (Fig. 2, E and F). Thus, the feeding-induced control of ISC size is region specific and the spatial distribution of ISC growth corresponds to the distribution of EB accumulation upon feeding (Fig. 1K).

To understand how the region-specific ISC growth is regulated, we investigated the role of the mTORC1 signaling pathway, a known regulator of cell size (*17*). Midguts were analyzed for the mTORC1 target initiation factor 4E–binding protein phosphorylation (p4EBP) that responded to genetic inhibition of mTORC1 through regulatory-associated protein of mTOR (Raptor) RNA interference (RNAi) expression in ISCs (fig. S2, A and B) (*27*). We monitored the levels of p4EBP in regions R1, R2, R4, and R5, as well as the region borders flanking R3. Consistent with its role as cellular nutrient sensor, mTORC1 signaling showed increased activity in response to feeding in all intestinal regions (Fig. 2G). However, the cellular distribution of mTORC1 activity displayed notable regional heterogeneity. In the R1 region and in the borders flanking R3, p4EBP signal is mainly detected in polyploid ECs, while, in R4 and R5, p4EBP signal is high in the small nucleus cell population (Fig. 2G). In R2, p4EBP signal is mixed, showing signal in both large and small cells. Quantification of the intensity signal confirmed higher ISC p4EBP levels in R2 and R4 regions compared to R3-R4 border in refed animals (Fig. 2H). To explore the functional importance of mTORC1 signaling in the ISCs, we depleted mTORC1 activity by RNAi knockdown of Raptor using the ISC-specific esg-Gal4^ts^, Su(H)GBE-Gal80 driver and measured nuclear area from ISCs. ISCs of fed animals with Raptor RNAi were significantly smaller without a significant change in the main midgut region lengths or large nucleus area when compared to controls (Fig. 2I and fig. S2, C and D). As expected, the effect of Raptor RNAi was the strongest in R2 and R4 regions and weaker in the borders flanking R3 (Fig. 2I). Furthermore, superphysiological activation of mTORC1 in ISCs by knocking down TSC1, a component of the negative regulator of mTORC1 (*28*), led to increased ISC size in the R4 region (fig. S2, E and F). In conclusion, ISCs display a region-specific increase in size as an immediate response to feeding, which is controlled by mTORC1 activation.

### Cell cycle–specific regulation of ISC mTORC1 signaling

**Fig. 3.**
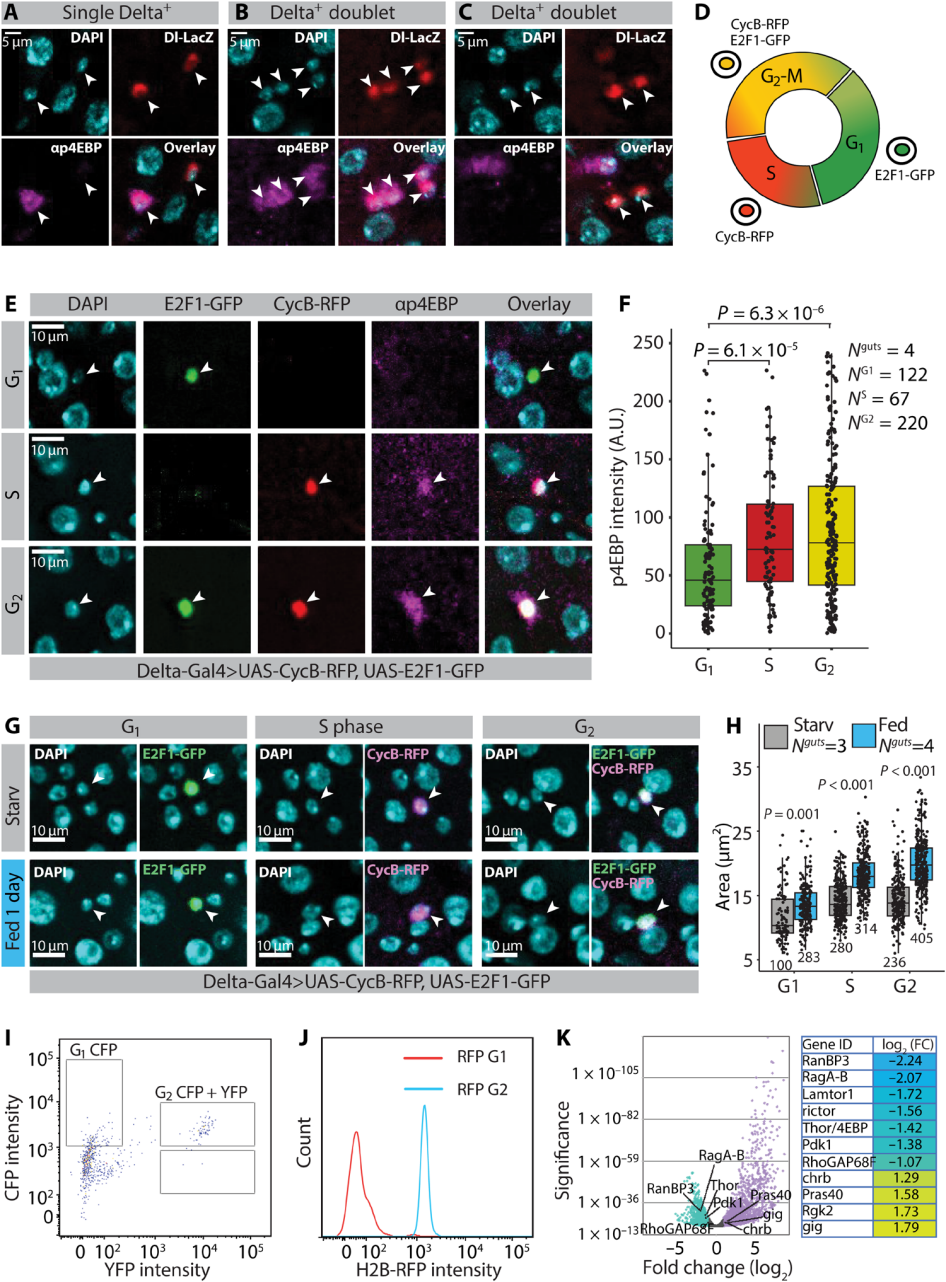
ISC mTORC1 activity is regulated in a cell cycle–dependent manner. (**A** to **C**) mTORC1 activity is heterogeneous between ISCs. Representative images of single (A) and doublet [(B) and (C)] Delta^+^ cells stained with α-p4EBP (magenta), α-β-galactosidase (red), and DAPI (cyan). Images are from the R4b region of Delta-LacZ bearing female flies. Arrowheads point to Delta^+^ nuclei. (**D**) Schematic of the FUCCI system. (**E** and **F**) mTORC1 activity is elevated in the S and G_2_-M. (E) Representative images from midguts of fly-FUCCI flies stained with α-p4EBP (magenta) and DAPI (cyan). Arrowheads point to Delta^+^ nuclei. (F) p4EBP intensity from G_1_, S, and G_2_ ISCs. (**G** and **H**) ISCs of the fed animals are larger compared to the starved animals at all cell cycle phases. (G) Representative images of G_1_, S, and G_2_-M ISCs from midguts of fly-FUCCI flies kept in either starvation or holidic diet. DAPI (cyan), E2F1-GFP (green), and CycB-RFP (magenta). Arrowheads point to Delta^+^ nuclei. (H) Quantification of the experiment depicted in (G). *N*^guts^ and *N*^cells^ are indicated in the figure panel. (**I** to **K**) Gene expression profiling of G_1_ versus G_2_-M ISCs shows differential expression (DE) of mTORC1 regulators. (I) Gating strategy to sort G_1_ (CFP only) and G_2_ (CFP + YFP) cells from ISC-specific esg-Gal4^ts^, Su(H)GBE-Gal80–driven CFP::E2F1/YFP::NLS-CycB Fly-FUCCI midguts. (J) An extra gating check was made by measuring the H2B::RFP intensity for the two different populations. Two clear peaks were detected, G_1_ (red) and G_2_ (blue). (K) enrichment and depletion of mTOR-associated genes in G_1_ stem cells. Positive mTORC1 regulators (*ragA-B*, *Lamtor1*, and *Pdk1*) are depleted in G_1_ ISCs, whereas negative regulators (*gig*/*TSC2*, *PRAS40*, and *charybdis*) are up-regulated in G_1_ ISCs. *P* values in (F) and (H) were obtained by two-way analysis of variance (ANOVA) followed by Tukey’s test. See also fig. S3 and table S2.

To further analyze mTORC1 activity in the diploid cell population, we explored the p4EBP pattern specifically in the Delta^+^ ISC population. The mTORC1 activity was heterogeneous among ISCs. We identified both single ISCs and ISC-ISC doublets, either positive or negative for the mTORC1 marker p4EBP (Fig. 3, A to C). This heterogeneity led us to test whether mTORC1 activity depends on the cell cycle phase. We used Delta-Gal4 to express the fluorescent ubiquitination-based cell cycle indicator (FUCCI) reporter (fly-FUCCI) (*29*) in the ISCs, which allowed us to analyze 4EBP phosphorylation during growth phase 1 (G_1_), synthetic phase (S), and growth phase 2 (G_2_)–mitosis (M) separately (Fig. 3D). This analysis revealed that mTORC1 activity is low in G_1_ and gets gradually elevated in S and G_2_-M (Fig. 3, E and F), consistent with the observed heterogeneity of mTORC1 activity in the total ISC population. We also used the fly-FUCCI system to analyze feeding-induced ISC growth in different cell cycle phases. Our data show that ISCs of the fed animals were significantly larger compared to the starved animals at all cell cycle phases and the difference between the size distributions gradually increased in S and G_2_-M, consistent with the mTORC1 activity (Fig. 3, G and H). Thus, the mTORC1-dependent ISC growth is gradually activated, while the cell cycle progresses toward mitosis. Last, we monitored the regional distribution of progenitor cells in G_1_, S, and G_2_ and observed an enrichment of S and G_2_ at R2 and R4 regions in midguts of fed flies corresponding with the regions of activated mTORC1 signaling in ISCs (fig. S3, A to C).

To better understand the cell cycle–specific regulation of mTORC1 activity in the ISCs, we performed gene expression profiling in G_1_ versus G_2_-M, using fluorescence-activated cell sorting of FUCCI-marked ISCs. Briefly, we combined a upstream activating sequence (UAS)–driven CFP::E2F1/YFP::NLS-CycB fly-FUCCI with His2AV::mRFP (to mark all nuclei) and used the ISC-specific esg-Gal4^ts^, Su(H)GBE-Gal80 line to restrict expression to ISCs. The cyan fluorescent protein (CFP) and yellow fluorescent protein (YFP) signals were used to separate G_1_ and G_2_-M ISCs, and total RNA was isolated from both populations (Fig. 3I). Analysis of the His2AV-mRFP fluorescence signal in G_1_ and G_2_-M populations showed a clear distinction of these populations based on nuclear His2AV levels (Fig. 3J). Differential expression (DE) analysis identified 1690 genes up-regulated and 1235 genes down-regulated in G_1_ ISCs compared to G_2_-M ISCs. Among the down-regulated genes, we found several positive regulators of mTORC1-activity, such as* ragA-B*, *Lamtor1*, and *Pdk1*. Conversely, we found G_1_-specific up-regulation of several negative mTORC1 regulators, such as *charybdis*, *gigas* (*gig*/*TSC2*), and *PRAS40* (Fig. 3K and table S2) (*30*). Together, our comparison of the transcriptomes of ISCs in G_1_ and G_2_-M of the cell cycle suggests that the ISCs in G_1_ keep mTORC1 in an inhibited state, which is released upon cell cycle progression to G_2_-M.

### ISC mTORC1 signaling is associated with asymmetric ISC-EB cell pairs

**Fig. 4.**
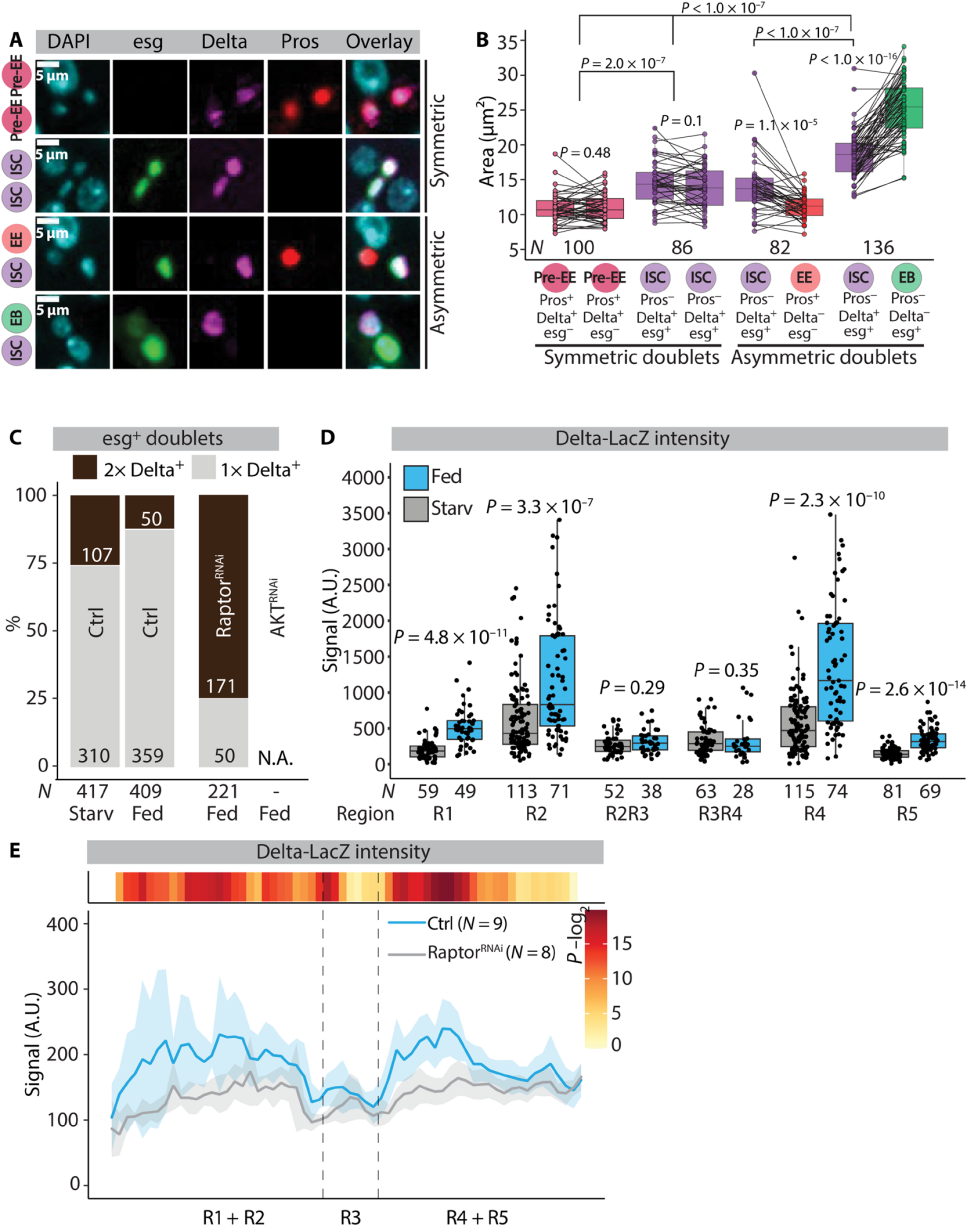
ISC size correlates with progenitor doublet type. (**A** and **B**) ISC size is associated with progenitor doublet type. (A) Representative images of symmetric (top) and asymmetric (bottom) doublet types from midguts of female flies of genotype esg-Gal4^ts^>UAS-GFP, Delta-LacZ kept in holidic diet and stained with α-β-galactosidase (magenta), α-Prospero (red), and DAPI (cyan). (B) Quantification of doublet nucleus area from the experiment depicted in (A). *N*^doublets^ are indicated in the figure panel. Pooled data from *N* = 4 midguts. (**C**) Number of Delta^+^ esg^+^ cell doublets from female midguts of esg-Gal4>fly-FUCCI, Delta-LacZ (Ctrl) in combination with Raptor-RNAi or Akt-RNAi. Pooled data from *N*^starved^ = 4 and *N*^fed^ = 4 (control) and *N*^*f*ed^ = 5 (Raptor-RNAi) midguts from the R4b region. *N*^doublets^ are indicated in the figure panel. Experimental design depicted in fig. S4A. (**D**) Delta-LacZ intensity measurements (α-β-galactosidase immunostaining) from R1, R2, R4, and R5 and borders flanking R3 from midguts of female flies kept in either starvation or holidic diet. Pooled data from *N*^starved^ = 6 and *N*^fed^ = 5 midguts. *N*^cells^ are indicated in the figure panel. Experimental design depicted in Fig. 1A. (**E**) Delta expression is mTORC1 dependent. Regional quantification of Delta-LacZ intensity (α-β-galactosidase immunostaining) from midguts of female flies in holidic diet of genotype esg-Gal4^ts^, Su(H)GBE-Gal80, Delta-LacZ (Ctrl) in combination with Raptor-RNAi. *N*^guts^ are indicated in the figure panel. Experimental design depicted in Fig. 1A. *P* values in (B) were obtained by paired *t* test (testing within doublets) or two-way ANOVA followed by Tukey’s test (testing between doublets). *P* values in (D) were obtained by Wilcoxon rank sum test with multiple testing correction (FDR < 0.05). *P* values in (E) were obtained by Wilcoxon rank sum test using continuity and FDR correction (FDR < 0.05). See also fig. S4.

What is the role of mTORC1 signaling in ISCs? On the basis of the regional correlation with cell-type patterning, ISC size, and mTORC1 activity, we hypothesized that cell size might correlate with ISC differentiation. ISCs can divide either symmetrically to produce two ISCs or asymmetrically into one ISC and one EB, which undergoes rapid growth during differentiation toward EC fate (*2*, *9*). In addition to EBs, ISCs can differentiate into small diploid EE cells. EE cells can arise through three mechanisms: by asymmetric ISC-EE division, symmetric ISC division producing two EE cells, or by direct ISC to EE differentiation (*31*). Notably, Prospero^+^ EEs do not arise through a Su(H)^+^ EB intermediate in the adult posterior midgut (*31*). Differentiation of EBs is regulated by Notch signaling dependent on physical cell-cell contact between the ISC and EB (*6*). Thus, we analyzed the size of ISCs in symmetric versus asymmetric esg^+^ progenitor doublets. These doublets arise either through ISC division or, to a lesser extent, through association of non-sibling cells (*32*). First, we quantified ISC nuclei sizes from symmetric Delta^+^ Delta^+^ and asymmetric Delta^+^ Delta^−^ cell doublets (Fig. 4, A and B). To confirm that the Delta^+^ Delta^−^ cell doublets mostly represent asymmetric ISC-EB cell pairs, we compared the size of their nuclei. As expected, the Delta^+^ Delta^−^ cell doublets show substantial size asymmetry, the Delta^−^ (expected EB) being significantly larger compared to its Delta^+^ (ISC) progenitor partner (Fig. 4, A and B). The mean ISC size in symmetric Delta^+^ Delta^+^ doublets was significantly smaller compared to the ISCs in asymmetric Delta^+^ Delta^−^ doublets, suggesting that the larger ISCs associate with a pair that undergoes EB differentiation (Fig. 4, A and B). In addition, the size of the Delta^+^ ISC in asymmetric ISC-EE doublets and symmetric Delta^+^ prospero^+^ pre-EE doublets were also smaller compared to the ISCs in the asymmetric Delta^+^ Delta^−^ doublet (Fig. 4, A and B). Together, in ISC containing cell pairs, large ISCs mostly associate with neighboring cells that differentiate toward the EB fate, whereas smaller ISCs are found in symmetric Delta^+^ Delta^+^ doublets and in asymmetric doublets associated with cells expressing the EE cell marker Prospero.

Next, we scored symmetric (Delta^+^ Delta^+^) versus asymmetric (Delta^+^ Delta^−^) esg^+^ progenitor doublets from midguts of starved and fed animals. Consistent with the model of nutrient availability in favoring the formation of asymmetric ISC-EB doublets, the proportion of symmetric Delta^+^ Delta^+^ doublets was higher in midguts of starved animals compared to the fed ones (Fig. 4C and fig. S4A). To directly address the causal relationship between ISC mTORC1 activity and frequency of symmetric versus asymmetric doublet formation, we monitored progenitor doublets from flies expressing RNAi against Raptor driven by the esg-Gal4 driver. As the knockdown of Raptor did not prevent ISC division, we were able to address the role of mTORC1 activity in formation of symmetric versus asymmetric doublets (fig. S4B). Consistent with the indirect evidence associating increased ISC size with asymmetric ISC-EB doublet formation, inhibition of mTORC1 signaling by the esg-Gal4 driver reduced the relative amount of asymmetric Delta^+^ Delta^−^ doublets (Fig. 4C and fig. S4A). This was in contrast to the knockdown of Ser and Thr kinase Akt, a well-known transducer of the PI3K signaling (*33*), which strongly inhibited the feeding-induced increase in numbers of ISC-derived cells as well as doublet formation, consistent with the important role of PI3K/Akt signaling in ISC proliferation (Fig. 4C and fig. S4B) (*9*, *11*). Thus, high mTORC1 activity is necessary for asymmetric ISC-EB doublet formation.

### ISC mTORC1 signaling promotes Delta expression

Because the EB fate determination is controlled by the activity of Delta-Notch signaling (*6*, *34*, *35*), we wanted to explore the relationship between ISC mTORC1 activity and Delta expression. To test the correlation between ISC size and Delta expression, we measured regional Delta-LacZ intensity in starved and fed animals. Delta expression was highly region specific, being low in the regions with small ISCs (R1, borders flanking R3 and R5) and being high in the regions with larger ISCs (R2 and R4) (Fig. 4D). Furthermore, the expression of Delta was diet-dependent, being most strongly activated by feeding in R2 and R4 and insensitive to feeding in borders flanking R3 (Fig 4D). Given the strong regional correlation with Delta expression, ISC size, and differentiation, we asked whether Delta expression is mTORC1 dependent. Raptor knockdown in ISCs significantly reduced Delta-LacZ intensity in R2 and R4 and less in the borders flanking R3 (Fig. 4E). Moreover, activation of mTORC1 signaling by ISC-specific knockdown of TSC1 led to increased Delta-LacZ signal intensity (fig. S2, E and G). Together, our results are consistent with a model that large ISCs with high mTORC1 activity express high levels of Delta, which directs differentiation toward EB fate in a region-specific manner.

### ISC mTORC1 inhibits EE cell differentiation

**Fig. 5.**
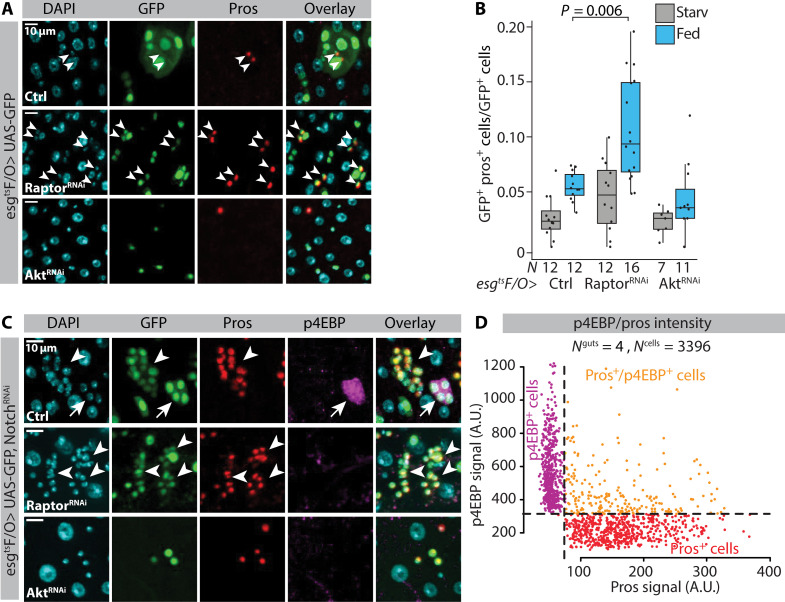
mTORC1 directly inhibits EE cell fate. (**A**) Representative images of female midgut R4b regions of the genotype esg^ts^F/O>UAS-GFP (Ctrl) in combination with Raptor-RNAi or Akt-RNAi kept in holidic diet and stained with α-Prospero (red) and DAPI (cyan). Arrowheads point to pros^+^ esg^+^ nuclei. Experimental design depicted in Fig. 1A. (**B**) Quantification of the relative number of total GFP^+^ Pros^+^ cells (normalized to total GFP^+^ cell numbers) from the experiment depicted in (A). Quantifications are performed from the R4b region. *N*^guts^ are indicated in the figure panel. Pooled data from two independent experiments. (**C**) Representative images from fed female midgut R4b regions of the genotype esg^ts^F/O>UAS-GFP, Notch-RNAi in combination with Raptor-RNAi or Akt-RNAi stained with α-Prospero (red), α-p4EBP (magenta), and DAPI (cyan). Arrowheads point to esg^+^ pros^+^ p4EBP- cell clusters. Arrows in the top panel point to esg^+^ pros^−^ p4EBP^+^ cell cluster. Experimental design depicted in Fig. 1A. (**D**) High mTORC1 activity and EE cell identity are mutually exclusive. α-Prospero and α-p4EBP intensity signal dependency from GFP^+^ nuclei from the experiment depicted in (C). Quantifications are performed from the genotype esg^ts^F/O>UAS-GFP, Notch-RNAi from the R4c-R5 regions. *P* values in (B) were obtained by Wilcoxon rank sum test.

ISCs with low Delta expression can differentiate into the EE fate through a Prospero^+^ pre-EE state (*31*, *36*). Consistently, we noticed that Delta^+^ Prospero^+^ pre-EE cells were expressing significantly less Delta as compared to the Delta^+^ Prospero^−^ ISCs (fig. S4C). These pre-EE cells were also significantly smaller, compared to the rest of the Delta^+^ cell population (Fig. 4B). We next asked whether mTORC1 also actively inhibit EE cell differentiation. Knockdown of Raptor by the esg^ts^F/O driver led to significantly increased number of EE cells in the R4 region compared to the control under fed conditions (Fig. 5, A and B). Elevated EE differentiation upon mTORC1 inhibition might reflect direct inhibition of EE differentiation by mTORC1 or to be an indirect consequence of reduced EB differentiation. To distinguish between these alternatives, we investigated the relationship between mTORC1 signaling and EE cell differentiation in the context of *Notch* LOF, which inhibits ISC differentiation toward the EB lineage (*34*, *35*). *Notch* LOF leads to the formation of proliferative endocrine progenitor tumors with a mixed population of Prospero^+^ and Prospero^−^ cells (*37*). We stained *Notch* LOF tumors in fed conditions with the mTORC1 activity marker anti-p4EBP, which led to the identification of p4EBP^−^ and p4EBP^+^ subclusters (Fig. 5C). Notably, Prospero^+^ cells were found in p4EBP^−^ clusters, while the p4EBP^+^ cell clusters were devoid of Prospero expression (Fig. 5D). The strong anticorrelation between mTORC1 activity and Prospero expression implied that high mTORC1 signaling directly inhibits EE cell fate. This was the case, as Raptor knockdown in the *Notch* LOF clones abolished the Prospero^−^ p4EBP^+^ clusters, turning the clusters homogenous for Prospero^+^ cells (Fig. 5C). In contrast, knockdown of Akt completely prevented the tumorous growth of the *Notch* LOF clones (Fig. 5C), consistent with its antiproliferative effect (Fig. 4C and fig. S4B). In conclusion, our data suggest that physiological nutrient–regulated mTORC1 signaling directly inhibits ISCs from differentiating to EE cells.

### Intermittent fasting protects from aging-induced decline of intestinal nutrient adaptation

**Fig. 6.**
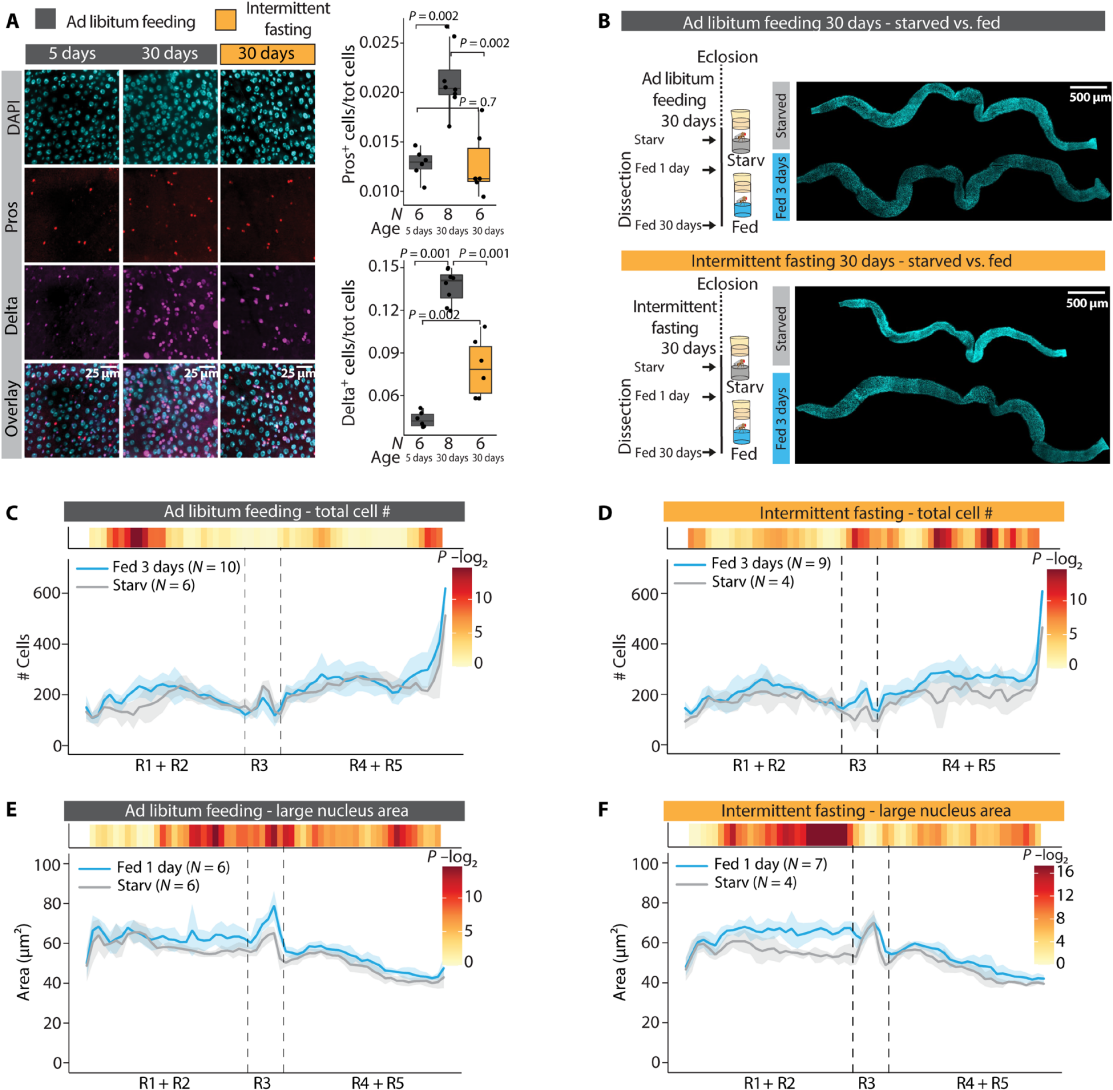
Intermittent fasting protects from aging induced decline in midgut nutrient adaptation. (**A**) Lifelong intermittent fasting reduces the accumulation of EE cells and ISCs in aging midgut. Representative images of α-Prospero and α-β-galactosidase-stained midguts from the R4b region of 5 day and 30-day old female esg-Gal4^ts^, UAS-GFP, Delta-LacZ flies, and quantification of total Prospero^+^ and Delta^+^ cells from the same region. The intermittent-fasted flies were at holidic diet at the time of harvesting midguts. Experimental design depicted in fig. S4A. (**B**) Experimental design used to obtain data in (B) to (F) and representative images of starved and refed midguts after 30 days of ad libitum feeding or intermittent fasting. (**C** and **D**) Regional quantification of starved versus 3 days refed midgut total cell numbers along the A/P axis after 30 days of ad libitum feeding (C) or intermittent fasting (D). (**E** and **F**) Regional quantification of starved versus 1 day refed midgut large nucleus area along the A/P axis after 30 days of ad libitum feeding (E) or intermittent fasting (F). *P* values in (A) were obtained by Wilcoxon rank sum test with multiple testing correction (FDR < 0.05). *P* values in (C) to (F) were obtained by Wilcoxon rank sum test using continuity and multiple testing correction (FDR < 0.05). See also fig. S5.

Aging is known to cause accumulation of mis-differentiated cells, leading to progressive loss of epithelial homeostasis (*38*, *39*). Nutrients are known to modulate this process because repeated feeding-fasting cycles (intermittent fasting) were previously shown to improve gut barrier function and to suppress ISC hyperproliferation in aged flies (*40*). However, it is not known how aging and the protective dietary interventions affect the mechanisms of intestinal nutrient adaptation. Thus, we compared the intestines of aged ad libitum–fed flies to flies of the same age exposed to lifelong intermittent fasting (fig. S5A). As reported previously, we found that the midguts of aged ad libitum–fed flies accumulate Prospero^+^ and Delta^+^ cells (Fig. 6A) (*39*). In the midguts of intermittent-fasted flies, the abnormal accumulation of Prospero^+^ and Delta^+^ cells was significantly suppressed, while the total midgut cell number remained unaffected (Fig. 6A and fig. S5, B and C).

**Fig. 7.**
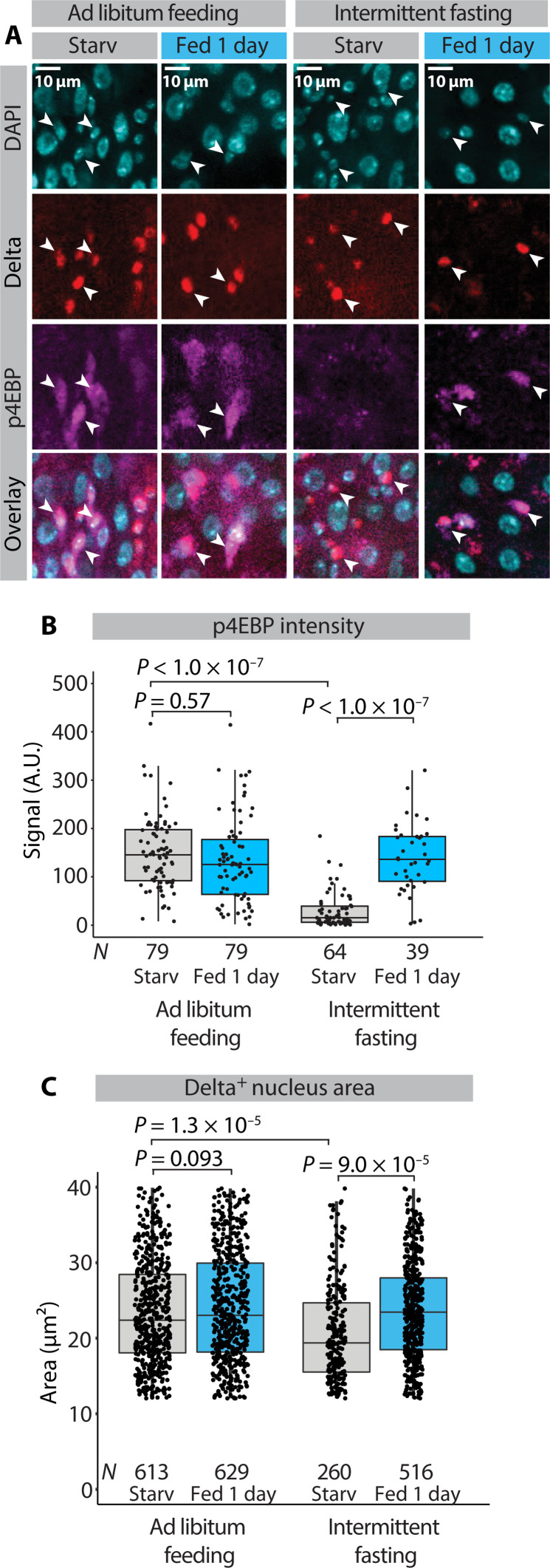
Intermittent fasting protects ISCs from aging induced decline in nutrient sensing. (**A**) Representative images of α-p4EBP– and α-β-galactosidase–stained midguts from the R4b region of aged ad libitum–fed or intermittent-fasted female esg-Gal4^ts^, UAS-GFP, Delta-LacZ flies subjected to starvation-refeeding cycle. Arrowheads point to Delta^+^ nuclei. Experimental design depicted in Fig. 6B. (**B**) Quantification of p4EBP intensity signal from Delta^+^ nuclei from the experiment depicted in (A). Pooled data from *N*^starved^ = 3 and *N*^fed^ = 3 (ad libitum feeding) and *N*^starved^ = 3 *N*^fed^ = 3 (intermittent fasting) midguts from the R4b region. *N*^cells^ are indicated in the figure panel. (**C**) Quantification of Delta^+^ nucleus area from the experiment depicted in (A). Pooled data from *N*^starved^ = 6 and *N*^fed^ = 6 (ad libitum feeding) and *N*^starved^ = 4 and *N*^fed^ = 7 (intermittent fasting) midguts from the R4b region. *N*^cells^ are indicated in the figure panel. *P* values in (B) were obtained by two-way ANOVA followed by Tukey’s test. *P* values in (C) were obtained by Wilcoxon rank sum test with multiple testing correction (FDR < 0.05).

We then asked how intermittent fasting affects the adaptive midgut size regulation of aged flies. To this end, we exposed the old ad libitum–fed flies to a starvation-refeeding cycle and compared the intestinal adaptation response to the intermittent-fasted flies of the same age. In the ad libitum–fed flies, the starvation-refeeding–mediated regulation of midgut size was very limited, showing impaired reduction of midgut size upon fasting (Fig. 6B). In contrast, the midguts of aged intermittent-fasted flies much better maintained their ability to regulate midgut size in response to the starvation-refeeding cycle (Fig. 6B). In line with this observation, refeeding-induced changes in cell number and cell growth were very modest in the old ad libitum–fed flies when compared to the intermittent-fasted flies (Fig. 6, C to F). This implies that the adaptive cellular responses that control midgut size in response to nutrition are lost upon aging, while intermittent fasting can delay this process. Last, we asked how ISC nutrient sensing is affected by aging. We found that the aged ad libitum–fed flies show constitutively high ISC mTORC1 activity and larger ISC size in starved condition, as compared to the intermittent-fasted flies (Fig. 7, A to C). These results show that the physiological nutrient–dependent regulation of mTORC1 signaling is lost in the ISCs of aged ad libitum–fed flies and better preserved in intermittent-fasted flies. Together, our results show that repeated feeding-fasting cycles help to maintain the ability to ISC nutrient sensing and intestinal nutrient adaptation upon aging.

## DISCUSSION

Organ-wide analysis of the *Drosophila* midgut nutrient adaptation identified regionally distinct patterns of EC growth, cell numbers, and cell-type distribution upon transition from a fasted to a fed state. Fed animals display activation of the mTORC1 nutrient sensing pathway in the ECs or ISCs in a region-specific manner. The ISC mTORC1 activity is coupled to cell cycle progression, being highest during S and G_2_-M phases. Consistent with the activation of mTORC1, ISC size is prominently increased already 1 day after the transition to a fed state. Large ISCs with high mTORC1 activity display high expression of the Notch ligand Delta and are associated with asymmetric ISC-EB doublets, thus promoting differentiation toward the absorptive lineage. Furthermore, mTORC1 activity inhibits differentiation toward the secretory EE lineage, even under conditions where differentiation to the absorptive lineage is prevented (*Notch* LOF). Thus, our results show that dynamic control of ISC mTORC1 signaling mediates nutrient-responsive cell fate determination, regulating regionalized ISC differentiation patterns during adaptive growth of the intestine.

Previous works have shown that feeding promotes *Drosophila* ISC proliferation and EC size as well as villi length and cellular turnover of the mouse small intestine (*2*, *4*, *10*, *24*, *41*, *42*). Our quantitative organ-wide analysis of the *Drosophila* midgut revealed substantial regional differences in the regulation of intestinal cell number and size. Moreover, our data uncovered dynamic changes in regional distributions of differentiated cells. What are the physiological reasons for the regional differences in the adaptive responses? ECs in distinct regions differ in their metabolic functions (*12*, *14*, *43*, *44*). Such differences might lead to locally different needs for optimal EC turnover rate, reflected to ISC proliferation/differentiation upon intestinal nutrient adaptation. In addition to ECs, EE cells can be divided into regionally distributed subtypes depending on the hormones that they express (*44*, *45*). It will be interesting to explore whether the observed increase in EE cell numbers in the middle parts of the midgut is associated with nutrient-responsive changes in specific EE cell subtypes. Mechanistically, we observed that transition to a fed state activated the mTORC1 signaling pathway throughout the whole intestine but with distinct local distributions between the ISCs versus ECs. How are the regional patterns of ISC mTORC1 activity achieved? As ISCs are located on the basal side of epithelium, their access to intestinal lumen-derived nutrients likely depends on their local interactions with the nutrient-absorbing ECs. In line with this idea is the finding that the metabolism of mouse ISCs is regulated by controlled exchange of nutrients with their neighboring cells (*46*). We observed mutually exclusive regional activation patterns of the mTORC1 between ISCs and ECs, which is consistent with a model that ECs might either activate a cell autonomous growth program or, alternatively, channel the mTORC1 activating nutrients to the neighboring stem cells, facilitating their growth. Furthermore, it has been shown that the EC-ISC contacts restrict ISC divisions through E-cadherin–mediated signaling and that enlarged ECs, through expression of a constitutively active insulin receptor, inhibit the proliferation of nearby ISCs (*10*, *47*). Moreover, an inverse correlation between ISC proliferative activity and the EC size was observed by screening for infection response in genetically different *Drosophila* lines (*48*). Future studies should be directed to resolve the role of local tissue environment in coordinating ISC proliferation, growth, and differentiation as well as the physiological roles of region-specific ISC regulation.

Previous work has demonstrated that mTORC1 contributes to activation of quiescent somatic stem cells (*21*), while prolonged superphysiological mTORC1 activity in stem cells results in loss of stemness and self-renewal as well as loss of EE cell fate (*18*, *19*, *49*). Our study advances the understanding on the role of stem cell mTOR signaling, causally linking its activity to cell fate regulation in the context of intestinal nutrient adaptation. Although we were not able to directly address the specific role of ISC size in mediating the downstream effects of mTORC1 signaling, the close correlation between ISC size and fate determination raises the possibility of a causal relationship. How could ISC size direct the outcome of fate decision? Notch signaling is the key determinant between the absorptive versus secretory lineages. The level of Delta expression dictates the future fate of dividing ISC, as high Delta-induced Notch activation promotes the absorptive lineage differentiation (*6*). We found that the expression of Delta is strongly activated in an mTORC1-dependent manner, particularly in regions displaying ISC growth and elevated number of EBs in response to feeding. While it remains unresolved how mTORC1 signaling influences Delta expression, it is tempting to speculate that cell size might affect the perception of niche-derived signals and, consequently, the fate of the dividing ISC. *Drosophila* ISCs reside basally in a space restricted by the neighboring ECs, the basement membrane, and the underlying muscle layer (*5*). The ISC contact with the basement membrane through integrin signaling is essential for the maintenance of stem cell identity (*6*, *50*). During nutrient-induced midgut growth, symmetric ISC divisions take place in a basal-to-basal orientation, whereas the asymmetric ISC-EB divisions in a basal-to-apical orientation (*2*). ISC growth–induced changes in the relative contact surface to the basement membrane could potentially alter the niche-derived signaling strength and, hence, the outcome of the daughter cell fate. In line with this idea, it was recently shown that ISC shape, which is linked to its surface to volume ratio, changed the strength of the niche-derived intracellular signaling activities of the ISC in mouse small intestine (*51*).

Earlier studies have shown that increased stem cell size is an aging factor (*20*, *52*), which is in line with our findings on nutrition-insensitive large size of ISCs in the ad libitum–fed aged animals. The observed constitutively high mTORC1 signaling in ISCs is consistent with an earlier study showing elevated mTORC1 signaling in ISCs of aged mice (*21*, *53*). Future studies should be directed to better resolve the molecular mechanism underlying the aging-related deregulation of mTORC1 signaling and its role in impaired dynamic control of ISC differentiation. Our findings that lifelong intermittent fasting helps aged animals to sustain the ability to control mTORC1 activity and adapt midgut size to changing nutrition may open previously unknown intervention possibilities to delay aging-related decline of tissue function.

## MATERIALS AND METHODS

### Drosophila stocks and husbandry

Fly stocks used in this study are as follows: w; esg-Gal4, Tub-Gal80^ts^, UAS-GFP ; UAS-Flp, Act>CD2>Gal4 (esg^ts^F/O) (*25*), w; esg-Gal4, Tub-Gal80^ts^, UAS-GFP (*52*), esg-Gal4, Tub-Gal80^ts^, Su(H)GBE-Gal80, 2xYFP (*31*), Delta-Gal4 (*26*), Delta-LacZ (Bloomington 11651), Gbe+Su(H)-lacZ [Su(H)-LacZ] (*53*), Raptor-RNAi (Bloomington 31529), Akt-RNAi (Bloomington 33615), Notch-RNAi (Bloomington 27988), UAS-GFP-E2F1; UAS-mRFP1-NLS-CycB (Fly-FUCCI) (*29*), UAS-Reaper (Bloomington 5824). Flies were maintained at 25°C, on medium containing agar 0.6% (w/v), malt 6.5% (w/v), semolina 3.2% (w/v), baker’s yeast 1.8% (w/v), nipagin 2.4%, and propionic acid 0.7%. In experiments using the temperature sensitive Gal80^ts^, flies were reared at +18°C and then kept at +29°C to inactivate the Gal80^ts^ protein allowing the UAS-Gal4–driven transgene expression. The exact timings of the dietary treatment and temperature switch in each experiment are provided in the figure panels.

### Dietary treatments

As experimental diet (referred to as fed), we used the holidic diet described previously (*54*). The starvation medium (referred to as starv) in all starvation experiments and during intermittent fasting was 2% sucrose (w/v) in medium containing agar 0.5% (w/v), nipagin 2.4%, and propionic acid 0.7%. The pH of the starvation medium was adjusted to meet the pH of the holidic diet (pH 6.8) with NaOH.

### Immunohistochemistry

For immunofluorescence staining, intestines were dissected in phosphate-buffered saline (PBS) and fixed in 8% paraformaldehyde for 3 hours. Tissues were washed with 0.1% Triton X-100 in PBS and blocked in 1% bovine serum albumin for 1 hour. Subsequently, tissues were stained with anti–β-galactosidase (1:400) (MP Biomedicals, catalog no. 0855976-CF), anti-Prospero (1:1000) (Developmental Studies Hybridoma Bank, MR1A), anti-p4EBP (Cell Signaling Technology, 2855) antibodies. The samples were mounted in Vectashield mounting medium with DAPI (Vector Laboratories).

### FACS and RNA sequencing

Gut dissection and FACS dissociation were performed as described previously (*55*) with the following modifications. A total of 80 to 100 guts per sample were dissociated in elastase (4 mg/ml) with pipetting 15× to 20× every 15 min for 1 hour at 28°C with shaking at 600 rpm. After dissociation, cells were filtered through a 40-μm filter and FACS-sorted on the basis of CFP/YFP and mRFP-signal. Three triplicate samples of 80 to 100 guts each were used to sort G_1_ and G_2_ cells into RNA isolation buffer. RNA was isolated using the Arcturus PicoPure RNA isolation kit (Thermo Fisher Scientific) and subsequently amplified using the RiboAmp HS-Plus kit (Thermo Fisher Scientific). Libraries were generated using the TruSeq Stranded mRNA Library Prep Kit (Illumina) and subsequently sequenced as 50–base pair single-end on an Illumina HiSeq2500. Reads were mapped using Bowtie and reads were counted using htseqcount. Tables of raw counts per gene/sample were analyzed with the R package DESeq2 for DE (*56*). G_1_ and G_2_ populations were compared with each other. Genes with an adjusted *P* value of <0.05 were considered to be differentially expressed. Volcano plots were generated by VolcaNoseR (*57*). The raw sequencing data are deposited into the Gene Expression Omnibus under accession number GSE222254.

### Microscopy and image processing

Fixed and immunostained midguts were mounted in between a microscope slide with 0.12-μm spacers and a coverslip, followed by imaging by the Aurox clarity spinning disc confocal microscope. Images were further processed by the ImageJ software and segmented by Stardist as previously described (*16*). Raw feature data were exported and used as input for LAM for further analysis (*16*). Intensities were measured as average voxel or pixel intensities from the segmented regions of interest.

### LAM analysis

Segmented midgut image data were used for border region analysis and quantitative regional analysis as previously described (*16*). Briefly, piecewise median lines, which we colloquially call vectors, are generated onto the two-dimensional spatial data matrix, and all the segmented image objects are projected onto the vector divided into user-defined number of bins. Consequently, LAM enables building of data matrices for bin-to-bin and windowed statistical comparisons. Border regions were detected by a multivariate analysis using localized changes in values of polyploid nucleus areas, nuclei nearest distances, and midgut widths.

### Statistical analysis

Statistical analyses were performed in R/Bioconductor. For parametric data, two-tailed *t* test, paired sample *t* test, or two-way analysis of variance (ANOVA) in conjunction with Tukey’s post hoc test was used. For nonparametric data, Kruskal-Wallis test and the Wilcoxon rank sum test with multiple testing correction, when applicable (false discovery rate < 0.05), were used. *P* values for the RNA sequencing experiment were calculated using the Wald significance test and adjusted with Benjamini and Hochberg correction for multiple testing. The exact test used in each experiment is indicated in the figure legend.
