# Supplementary material for: Stem cell mTOR signaling directs region-specific cell fate decisions during intestinal nutrient adaptation
Source: Sci Adv. 2024 Feb 9;10(6):eadi2671. doi: 10.1126/sciadv.adi2671 (PMC10857434; doi:10.1126/sciadv.adi2671)
Supplement: Supplementary file 1 — Figs. S1 to S5 Tables S1 and S2 [file sciadv.adi2671_sm.pdf]

Science Advances
AAAS

Supplementary Materials for

Stem cell mTOR signaling directs region-specific cell fate decisions during intestinal nutrient adaptation

Jaakko Mattila *et al.*

Corresponding author: Jaakko Mattila, jaakko.i.mattila@helsinki.fi; Ville Hietakangas, ville.hietakangas@helsinki.fi

Sci. Adv. **10**, eadi2671 (2024)
DOI: 10.1126/sciadv.adi2671

This PDF file includes:

Figs. S1 to S5
Tables S1 and S2

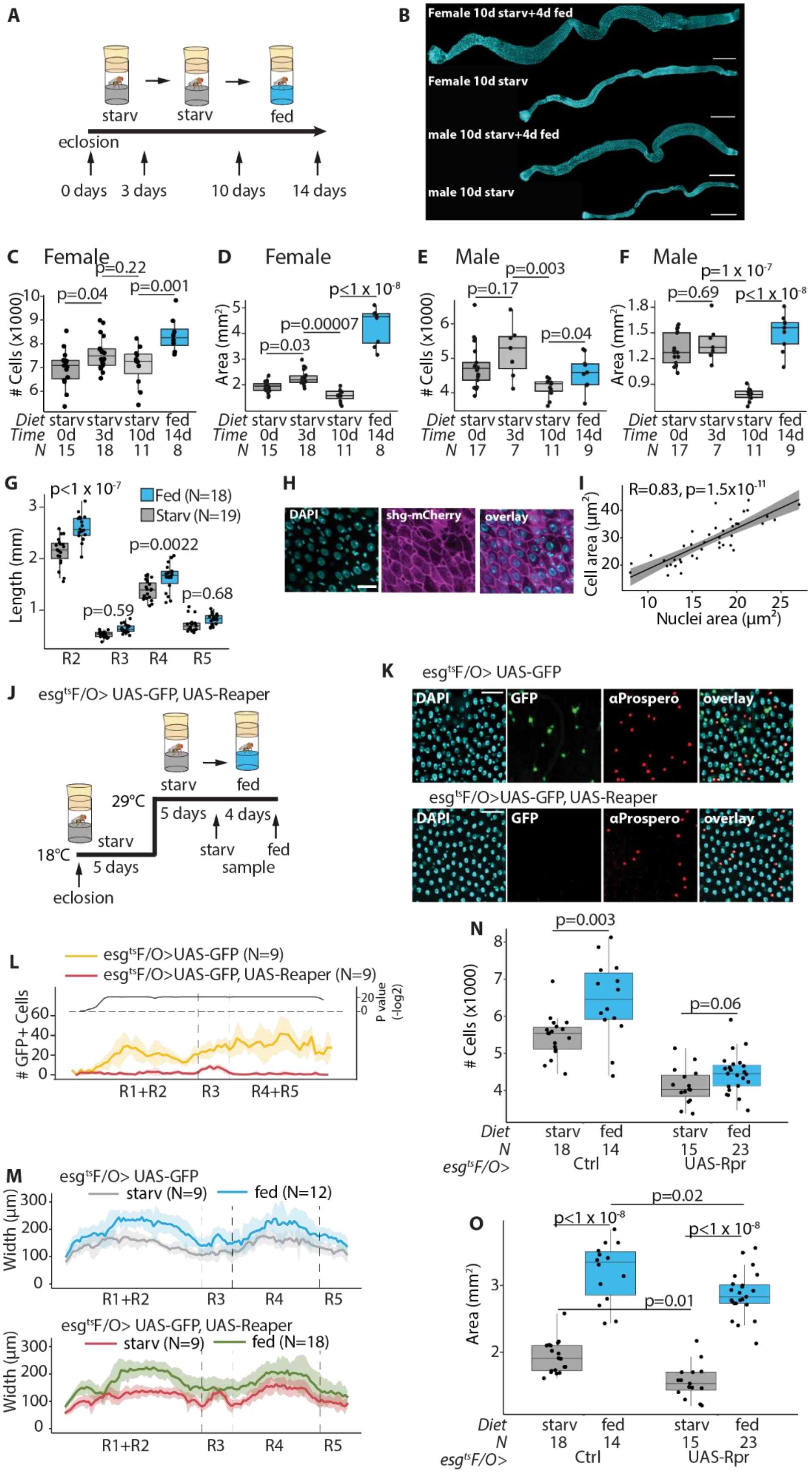
A
starv
starv
fed
eclosion
0 days
3 days
10 days
14 days
B
Female 10d starv+4d fed
Female 10d starv
male 10d starv+4d fed
male 10d starv
C
Female
p=0.22
p=0.04
p=0.001
# Cells (x1000)
Diet starv starv starv fed
Time 0d 3d 10d 14d
N 15 18 11 8
D
Female
p<1 x 10-8
p=0.00007
p=0.03
Area (mm2)
Diet starv starv starv fed
Time 0d 3d 10d 14d
N 15 18 11 8
E
Male
p=0.003
p=0.17
p=0.04
# Cells (x1000)
Diet starv starv starv fed
Time 0d 3d 10d 14d
N 17 7 11 9
F
Male
p=1 x 10-7
p=0.69
p<1 x 10-8
Area (mm2)
Diet starv starv starv fed
Time 0d 3d 10d 14d
N 17 7 11 9
G
p<1 x 10-7
Fed (N=18)
Starv (N=19)
p=0.0022
p=0.59
p=0.68
Length (mm)
R2 R3 R4 R5
H
DAPI
shg-mCherry
overlay
I
R=0.83, p=1.5x10-11
Cell area (µm2)
Nuclei area (µm2)
J
esgtsF/O> UAS-GFP, UAS-Reaper
starv
fed
29°C
5 days
4 days
starv
fed
sample
starv
18°C
5 days
eclosion
K
esgtsF/O> UAS-GFP
DAPI
GFP
αProspero
overlay
esgtsF/O>UAS-GFP, UAS-Reaper
DAPI
GFP
αProspero
overlay
L
esgtsF/O>UAS-GFP (N=9)
esgtsF/O>UAS-GFP, UAS-Reaper (N=9)
GFP+ Cells
P value (-log2)
R1+R2 R3 R4+R5
M
esgtsF/O> UAS-GFP
starv (N=9)
fed (N=12)
Width (µm)
R1+R2 R3 R4 R5
esgtsF/O> UAS-GFP, UAS-Reaper
starv (N=9)
fed (N=18)
Width (µm)
R1+R2 R3 R4 R5
N
p=0.003
p=0.06
# Cells (x1000)
Diet starv fed starv fed
N 18 14 15 23
esgtsF/O> Ctrl UAS-Rpr
O
p=0.02
p<1 x 10-8
p<1 x 10-8
p=0.01
Area (mm2)
Diet starv fed starv fed
N 18 14 15 23
esgtsF/O> Ctrl UAS-Rpr

Fig. S1.

Related to the main **Fig. 1.**

A-F) Midgut cell number and size are dynamically regulated by nutrients. **A)** Experimental design used to obtain data in panels B-F. Age matched, mated wild type (OregonR) females and males were kept at +25°C in starvation for ten days, and then shifted to the holidic diet for an additional 4 days. Midgut samples were obtained at 0, 3, 10 and 14 days. **B)** Representative images of female and male midguts at 10 days (starved) and 14 days (fed 4 days) time points. Scale bar is 200µm **C-F)** Quantification of female (**C**) and male (**E**) midgut cell numbers at the indicated time points. Quantified from one cell layer of a flattened, two-layer midgut. Hence the total midgut cell number is approximately 2x of the indicated value. Quantification of total surface area of a flattened female (**D**) and male (**F**) midgut at the indicated time points. **G)** Quantification of midgut region lengths in starved vs. fed female flies. Experimental design depicted in **Fig. 1A**. **H & I)** Midgut nuclear area and cell area correlate. **H)** Representative images of shg-mCherry (magenta) and DAPI (cyan) stained midguts. Scale bar is 20µm. **I)** Spearman correlation between nucleus (DAPI stained nuclei in G) and cell area (shg-mCherry -restricted area in H). **J-O)** Dietary nutrient induced midgut growth is not dependent on ISCs. **J)** Experimental design used to obtain data in panels **K-O**. **K)** Representative images of midgut R4b region from female flies of genotypes esg^{ts}F/O>UAS-GFP (Ctrl) or esg^{ts}F/O>UAS-GFP, UAS-Reaper. Scale bar is 50µm. **L)** Regional profile of GFP+ cell numbers at the time of dietary switch from starved to fed. Experimental design is depicted in **J**. Light yellow/red shading is the standard deviation. **M)** Width profile along the A/P axis of starved and fed midguts of Ctrl (upper panel) and ISC depleted flies (lower panel). Experimental design is depicted in **J**. Light blue/gray (top) and green/red (below) shading is the standard deviation. **N-O)** Total midgut cell numbers **(N)** and total midgut area **(O)** of control and ISC depleted flies in starved and fed condition. Quantified from one cell layer of a flattened, two-layer midgut. Hence the total midgut cell number is approximately 2x of the indicated value. P values in **C, E** and **N** were obtained by Wilcoxon rank-sum test with multiple testing correction (FDR<0.05). P values in **D**, **F**, **G** and **O** were obtained by two-way ANOVA followed by Tukey's test. P values in **L** were obtained by Wilcoxon rank-sum test using continuity and false discovery rate correction (FDR<0.05).

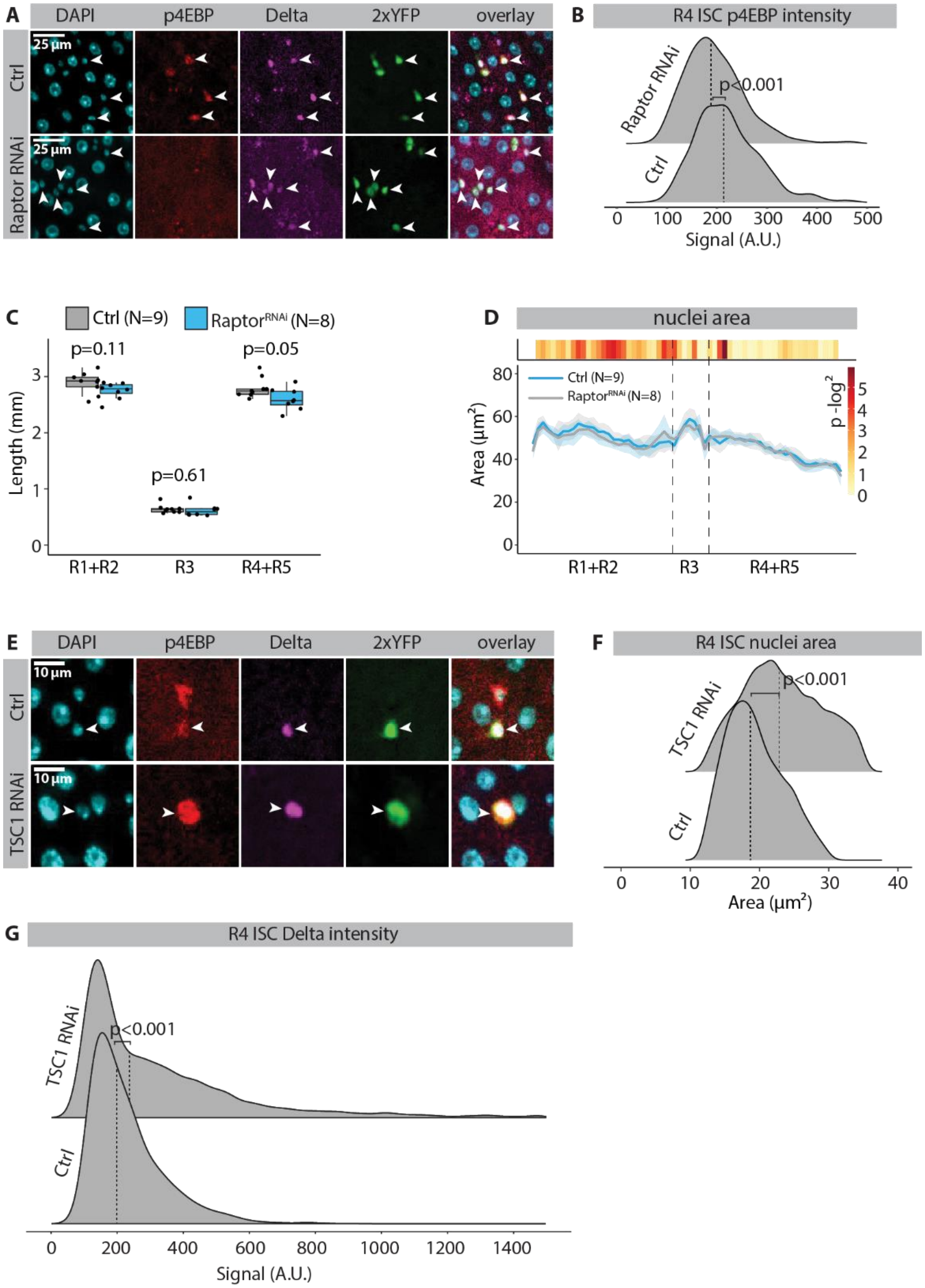

Fig. S2.

Related to the main **Fig. 2**.

A) Representative images of esg-Gal4^{ts}, Su(H)GBE-Gal80, UAS-2xYFP, Delta-LacZ (Ctrl) in combination with Raptor-RNAi from female midguts (R4b region) immunostained with α-β-Galactosidase (Delta) and α-p4EBP. Arrowheads point to Delta+ esg+ ISCs. Experimental design depicted in the main **Fig. 2I**. **B)** Quantification of α-p4EBP signal intensity from Delta+ cells from the experiment depicted in **A**. Quantification was performed from the R4bc region.

Dashed lines indicate median observations. **C)** Quantification of anterior (R1+R2), middle (R3) and posterior (R4+R5) region lengths from the experiment depicted in the main **Fig. 2I**. **D)** Regional quantification of the large nuclei area from the experiment depicted in the main **Fig 2I**. Light blue/gray shading is the standard deviation. **E)** Representative images of esg-Gal4^{ts}, Su(H)GBE-Gal80, UAS-2xYFP, Delta-LacZ (Ctrl) in combination with TSC1-RNAi from female midguts (R4b region) immunostained with α-β-Galactosidase (Delta) and α-p4EBP. Arrowheads point to Delta+ esg+ ISCs. Experimental design depicted in the main **Fig. 2I**. **F)** Quantification of ISC nuclei area from the experiment depicted in **E**. Quantification was performed from the R4bc region. Dashed lines indicate median observations. **G)** Quantification of ISC Delta signal intensity from the experiment depicted in **E**. Quantification was performed from the R4bc region. Dashed lines indicate median observations. P values in **B**, **F** and **G** were obtained by Wilcoxon rank-sum test. P values in **D** were obtained by Wilcoxon rank-sum test using continuity and false discovery rate correction (FDR<0.05). P values in **C** were obtained by two-tailed t-test.

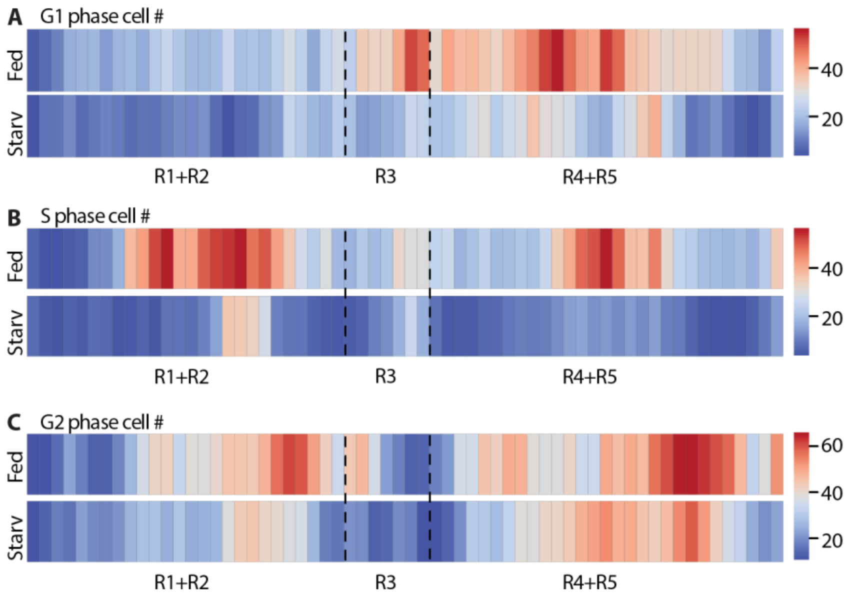

Fig. S3.

Related to the main **Fig. 3**.

A-C) Heatmaps showing regional distribution of the progenitor cell cycle phases in G1 **(A)**, S **(B)** and G2 **(C)** of the genotype esg-Gal4^{ts}>UAS-CycB-RFP, UAS-E2F1-GFP in starved and fed conditions. The number of G1 cells in **A** sums up ISCs and EBs since EBs possess only the G1 marker E2F1-GFP expression. Experimental design depicted in the main **Fig. 2I**.

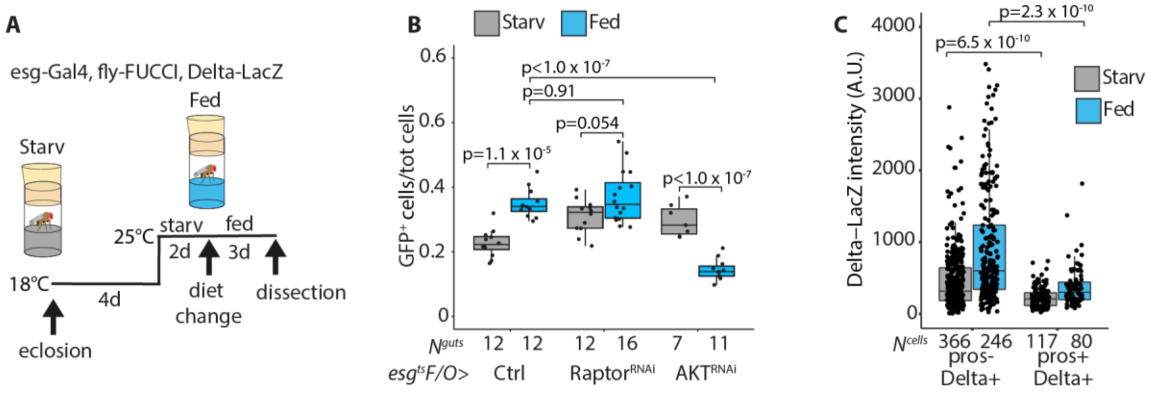

Fig. S4.

Related to the main **Fig. 4**.

A) Experimental design used to obtain data in the main **Fig. 4C**. Age matched, mated females of genotype esg-Gal4>UAS-CycB-RFP, UAS-E2F1-GFP, Delta-LacZ (Ctrl) in combination with Raptor-RNAi or Akt-RNAi, were aged for six days, and then shifted to the holidic diet for 3 days. **B)** Quantification of relative GFP+ cell number from the experiment depicted in the main **Fig. 4D** and **E**. Quantifications were performed from the R4-R5 regions from midguts of female flies of genotype esg^{ts}F/O>UAS-GFP (Ctrl) in combination with Raptor-RNAi or Akt-RNAi. N^{guts} are indicated in the figure panel. **C)** Quantification of Delta-LacZ signal intensity (α-β-Galactosidase immunostaining) from all α-Prospero positive EE cells from the experiment depicted in the main **Fig. 4F**. Measurements were from R1, R2, R4, R5 and borders flanking R3 from midguts of female flies kept in either starvation or holidic diet. Experimental design depicted in the main **Fig. 1A**. Pooled data from $N^{starved}$=6 and N^{fed}=5 midguts. N^{cells} are indicated in the figure panel. P values in **B** were obtained by two-way ANOVA followed by Tukey's test. P values in **C** were obtained by Wilcoxon rank-sum test with multiple testing correction (FDR<0.05).

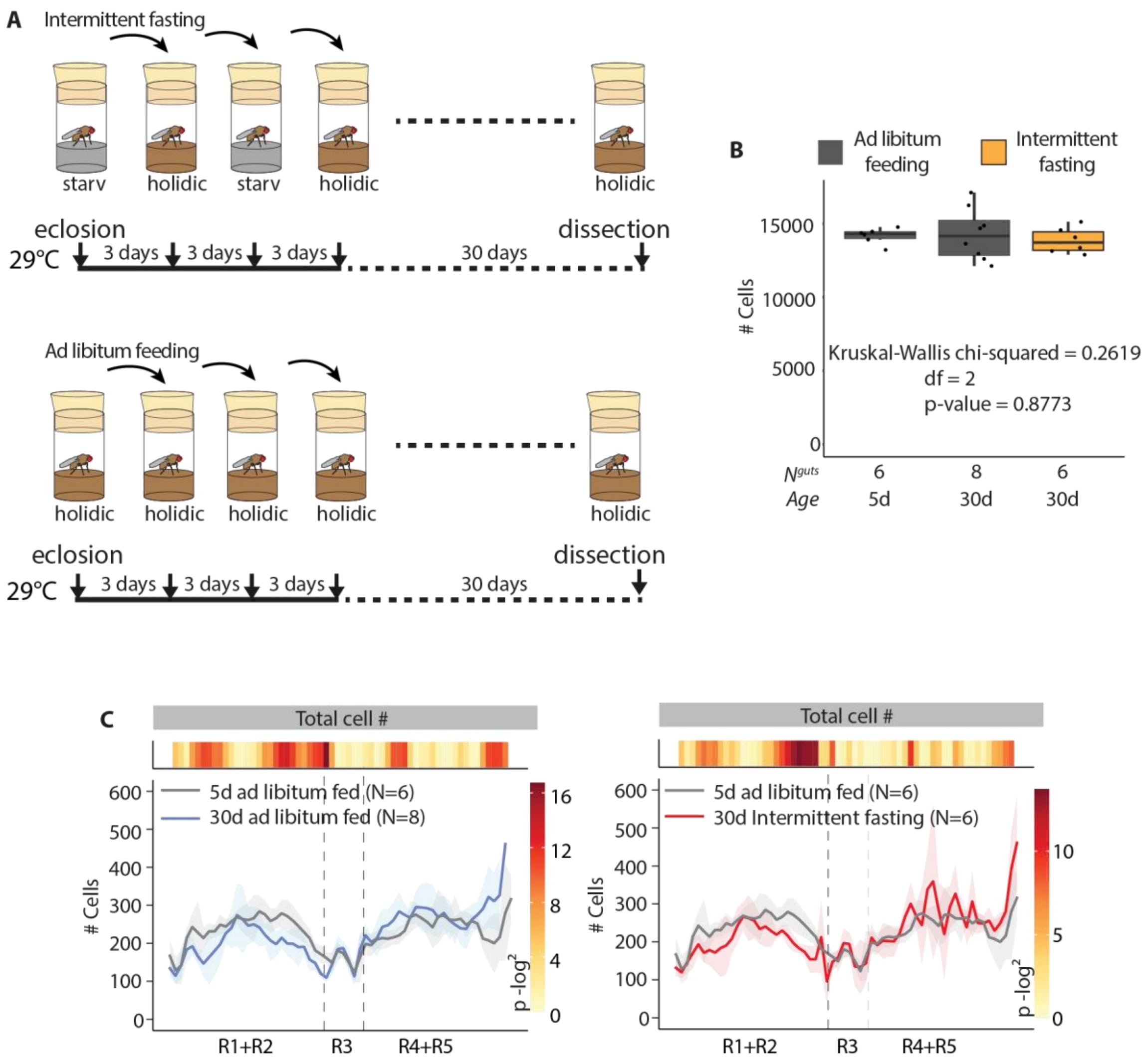

Fig. S5.
Related to the main **Fig. 6 and 7**.
A) Experimental design used for *ad libitum* fed and intermittent fasted flies. Used to obtain data in the main **Fig. 6 and 7**. Age matched females of genotype esg-Gal4^{ts}>UAS-GFP, Delta-LacZ were kept at 29°C in the presence of males for 30 days in holidic diet (*ad libitum* fed) or flipped to starvation after three days feeding period (intermittent fasting). **B)** Quantification of total cell numbers from the experiment depicted in the main **Fig. 6A**. N^{guts}, statistical test and significance are depicted in the figure panel. **C)** Total cell count comparisons along the midgut A/P axis between young (5d) and old (30d) *ad libitum* fed (left panel) and young (5d) and old (30d) intermittent fasted (right panel) female midguts. Light blue/gray and grey/red shadings are the standard deviation. P values in **C** were obtained by Wilcoxon rank-sum test using continuity and false discovery rate correction (FDR<0.05).

	R1	R2	R3	R4	R5
**Total cell #**	-	-	+	+	+
**EE #**	-	-	+	-	-
**EB #**	-	+	-	+	-
**GFP+ #**	-	-	+	+	-
**Large cell area**	+	+	+	-	-
**Large cell avg. distance**	+	+	-	-	-

Table S1. Summary of regional changes in midguts of fed flies compared to midguts of starved flies. Related to the main **Fig. 1**.

GeneID	p-adj
**RanBP3**	6,32E-10
**RagA-B**	2,72E-09
**Lamtor1/CG14184**	0,02431964
**rictor**	1,51E-06
**Thor/4EBP**	8,38E-07
**Pdk1**	3,66E-05
**RhoGAP68F**	0,0095798
**chrb**	0,00950493
**L/Pras40**	0,00131036
**Rgk2**	0,00096134
**gig**	0,01255781
**Mtor**	1,53E-09

Table S2. Adjusted p-values of mTOR regulator genes. Related to the main **Fig. 3K**.
